# Effects on Steroid 5-Alpha Reductase Gene Expression of Thai Rice Bran Extracts and Molecular Dynamics Study on SRD5A2

**DOI:** 10.3390/biology10040319

**Published:** 2021-04-11

**Authors:** Chiranan Khantham, Wipawadee Yooin, Korawan Sringarm, Sarana Rose Sommano, Supat Jiranusornkul, Francisco David Carmona, Wutigri Nimlamool, Pensak Jantrawut, Pornchai Rachtanapun, Warintorn Ruksiriwanich

**Affiliations:** 1 Department of Pharmaceutical Sciences, Faculty of Pharmacy, Chiang Mai University, Chiang Mai 50200, Thailand; ckhantham@gmail.com (C.K.); wipawadee.y@cmu.ac.th (W.Y.); supat.jira@cmu.ac.th (S.J.); pensak.j@cmu.ac.th (P.J.); 2 Cluster of Research and Development of Pharmaceutical and Natural Products Innovation for Human or Animal, Chiang Mai University, Chiang Mai 50200, Thailand; korawan.s@cmu.ac.th (K.S.); sarana.s@cmu.ac.th (S.R.S.); 3 Department of Animal and Aquatic Sciences, Faculty of Agriculture, Chiang Mai University, Chiang Mai 50200, Thailand; 4 Plant Bioactive Compound Laboratory (BAC), Department of Plant and Soil Sciences, Faculty of Agriculture, Chiang Mai University, Chiang Mai 50200, Thailand; 5 Departamento de Genética e Instituto de Biotecnología, Universidad de Granada, 18071 Granada, Spain; dcarmona@ugr.es; 6 Instituto de Investigación Biosanitaria ibs.GRANADA, 18014 Granada, Spain; 7 Department of Pharmacology, Faculty of Medicine, Chiang Mai University, Chiang Mai 50200, Thailand; wutigri.nimlamool@cmu.ac.th; 8 Cluster of Agro Bio-Circular-Green Industry, Faculty of Agro-Industry, Chiang Mai University, Chiang Mai 50100, Thailand; pornchai.r@cmu.ac.th; 9 School of Agro-Industry, Faculty of Agro-Industry, Chiang Mai University, Chiang Mai 50100, Thailand

**Keywords:** 5α-reductase, androgenetic alopecia, rice bran, tocopherol, SRD5A2, molecular dynamics, RD69, Tubtim chumphae

## Abstract

**Simple Summary:**

Dihydrotestosterone (DHT), the most potent androgen hormone, is an important aetiologic factor of androgenetic alopecia (AGA), or hair loss. Steroid 5-alpha reductases (SRD5As) increase DHT production in the scalp hair follicles, resulting in hair thinning and hair loss. Even though synthetic SRD5A inhibitors (finasteride and dutasteride) are effective in treating AGA, they cause adverse effects. This has led to an increased interest in alternative treatments from natural sources. The value of Thai rice bran has increased because several of its components may have use in AGA treatment. This study aimed to compare the suppression of the expression of *SRD5A* genes (type 1–3) exerted by several Thai rice bran extracts and investigate the interactional mechanism of their components towards SRD5A type 2. Tubtim Chumphae rice bran (TRB) had the highest sum of overall bioactive compounds. Among all extracts, the expression of *SRD5A* genes was suppressed by TRB as well as finasteride. In silico simulation showed that α-tocopherol had the greatest interaction with SRD5A type 2. Our findings identified α-tocopherol as the key bioactive in TRB; it could be developed as an anti-hair loss product.

**Abstract:**

Steroid 5-alpha reductases (SRD5As) are responsible for the conversion of testosterone to dihydrotestosterone, a potent androgen, which is the aetiologic factor of androgenetic alopecia. This study aimed to compare the *SRD5A* gene expression suppression activity exerted by Thai rice bran extracts and their components and investigate the interactional mechanism between bioactive compounds and SRD5A2 using molecular dynamics (MD) simulation. Bran of *Oryza sativa* cv. Tubtim Chumphae (TRB), Yamuechaebia Morchor (YRB), Riceberry (RRB), and Malinil Surin (MRB), all rice milling by-products, was solvent-extracted. The ethanolic extract of TRB had the highest sum of overall bioactive compounds (γ-oryzanol; α-, β-, and γ-tocopherol; phenolics; and flavonoids). Among all extracts, TRB greatly downregulated the expression of *SRD5A1*, *SRD5A2*, and *SRD5A3*; there were no significant differences between TRB and finasteride regarding *SRD5A* suppression. The linear relationship and principal component analysis supported that the α-tocopherol content was correlated with the *SRD5A* suppression exerted by TRB. Furthermore, MD simulation demonstrated that α-tocopherol had the highest binding affinity towards SRD5A2 by interacting with residues Phe118 and Trp201. Our findings indicate that α-tocopherol effectively downregulates the expression of *SRD5A* genes and inhibits SRD5A2 activity, actions that are comparable to standard finasteride. TRB, a source of α-tocopherol, could be developed as an anti-hair loss product.

## 1. Introduction

Androgenetic alopecia (AGA), characterised by the progressive replacement of terminal hair into small vellus hair, is generally known as a hereditary androgen-dependent disorder [1]. Although AGA is not a serious threat to health, it may impact the social and psychological well-being of an individual [2]. Different tissues, including hair follicles, require an optimal androgen concentration. High androgen levels can have deleterious effects on health [3]. Overexpression and intensive activity of steroid 5-alpha reductase (SRD5A) in scalp follicles have been shown to be involved in AGA development [4].

SRD5A comprises five members, SRD5A1, SRD5A2, SRD5A3, and the little characterised glycoprotein synaptic 2 (GSPN2) and GSPN2-like [5]. SRD5As are dihydronicotinamide adenine dinucleotide phosphate (NADPH)-dependent [6] and play a significant role in steroidogenesis by catalysing 4-ene-3-keto steroids into more active 5α-reduced derivatives, including the reduction of testosterone (T) to dihydrotestosterone (DHT) [7]. SRD5A1, SRD5A2, and SRD5A3 are encoded by separate genes: *SRD5A1*, *SRD5A2*, and *SRD5A3*, respectively. While these isozymes share sequence homology and show similar substrate preferences, they vary in biochemical properties, sensitivity to SRD5A inhibitors, physiological functions, and also tissue distribution [8]. DHT has a 5-fold higher affinity for the androgen receptor and a 10-fold greater potency for provoking androgen-sensitive genes compared with its precursor [9]. These androgen-sensitive gene products, transforming growth factor beta 1, interleukin 6, and dickkopf 1, have been identified as androgen-inducible negative mediators for AGA development [10].

*SRD5A* genes are expressed differently in androgen-responsive tissues, which include the adrenal glands, the testis, the placenta, and the skin. When translated, the proteins are located mainly in the endoplasmic reticulum membrane [11]. Beyond sexual functions, they have also been implicated in and influence the diverse biological activities of the skin [12]. Both SRD5A1 and SRD5A2 are well-characterised enzymes involved in AGA; they are expressed principally in skin and annexes, including hair follicles, sweat glands, and sebaceous glands [4]. The expression levels of *SRD5A1* and *SRD5A2* are higher in the frontal hair follicles in both men and women with AGA, a pattern that indicates they play a major role in AGA [13]. The level of *SRD5A2* expression is higher in dermal papilla cells (DPCs) from an AGA scalp than in DPC from other sites [1]. In contrast, *SRD5A3* is involved in protein *N*-glycosylation and shows slight or no potential to catalyse steroid substrates [8,14]. Interestingly, a previous study identified that the ratio of the total amounts of DHT to T is significantly depleted in *SRD5A3*-knockdown cells [15]. Moreover, tissue distribution analysis demonstrated that the expression level of *SRD5A3* is higher in peripheral tissues, including the skin, compared with *SRD5A1* and *SRD5A2* [16]. *SRD5A3* is overexpressed in prostate cancer [17], and several studies have proposed an association between prostate cancer and AGA [18,19]. A subsequent study also reported that a higher expression level of *SRD5A3* in plucked hair derived from AGA, suggesting its possible role in the pathogenesis of AGA [20].

To date, oral finasteride and dutasteride, SRD5A competitive inhibitors, have been approved to treat AGA. Finasteride specifically inhibits SRD5A2 as well as SRD5A3 but restrains less effectively SRD5A1. On the other hand, dutasteride exhibits great inhibitory effects against the three distinct types of SRD5A [5,16]. Despite their promising efficacy, these drugs are associated with several side effects, especially erectile dysfunction and loss of libido [21]. In recent years, several herbal extracts and their bioactive constituents have been implemented as an alternative treatment to promote hair growth or prevent hair loss [22].

Rice (*Oryza Sativa* Linn.) is used as a staple food by more than half of the globe [23]. Thailand is recognised as the biggest rice exporter and the fifth-biggest producer in the world [24]. Rice bran is an important by-product that is largely generated during the milling process [25]. In addition, it is a rich source of biologically active compounds, including γ-oryzanol, phytic acid, vitamin E isoforms (α-, β-, and γ-tocopherol), unsaturated fatty acids (such as oleic acid, linoleic acid, and γ-linolenic acid), and phenolic compounds (such as ferulic acid, gallic acid, and caffeic acid) [26]. The geographical origin and genetic diversity among rice varieties also influence the types, appearances, and bioactive content in rice bran [25].

Rice bran and its biomolecules have been shown to possess the potential for application as a treatment for hair loss and androgen-dependent disorders such as benign prostatic hyperplasia, hirsutism, and hypertrichosis [22]. Researchers suggest that specific aliphatic unsaturated fatty acids, especially oleic acid, linoleic acid, and γ-linolenic acid, inhibit the activity of SRD5A in androgen-responsive tissue [27]. In addition, various extracts from *Serenoa repensits*, *Thujae occidentalis*, *Cucurbita pepo*, and *Panax ginseng* inhibit SRD5A activity; these findings suggest that free fatty acids contribute to SRD5A inhibition [22]. Rice bran supercritical CO_2_ extracts and linoleic acid have been reported to suppress the messenger RNA (mRNA) expression of *SRD5A1* in cell lines [28]. Several studies have focused on the effect of plant extracts or their bioactive constituents, especially unsaturated fatty acids, on the inhibition of SRD5A activities, but only a few have focused on the gene expression levels [22].

Previous studies have investigated the inhibitory effects of rice bran extracts and their constituents on SRD5A1 and SRD5A2, but the effect on SRD5A3 is still unknown. Furthermore, the effect of other major components (vitamin E isoforms, phytic acid, and phenolic compounds) of rice bran extracts on the expression of *SRD5A* genes has not been thoroughly determined [29]. SRD5A2 is an important causative factor of AGA, and the aforementioned study was limited to SRD5A2. Moreover, the atomic-level mechanism between SRD5A2 and the bioactive compounds of rice bran extracts remains unclear. Molecular dynamics (MD) simulation can help to understand the binding mode of ligands towards SRD5A2, screen potential ligands, and accelerate candidate identification instead of the experimental method. With regards to these rationales, it is necessary to understand the biochemical actions of rice bran extracts and their bioactive compounds before converting this agricultural waste to a high-value-added anti-hair loss product. Hence, this study aimed to compare the *SRD5A* suppression exerted by four Thai rice bran varieties and their bioactive compounds and also to investigate the interactional mechanism at an atomic level of bioactive compounds in rice bran towards SRD5A2.

## 2. Materials and Methods

### 2.1. Reagents and Chemicals

Sulphorhodamine B (SRB); the Folin–Ciocalteu reagent; (−)-epigallocatechin gallate (EGCG); ferulic acid, phytic acid, gallic acid, oleic acid, linoleic acid, and γ-linolenic acid; γ-oryzanol; and α-, β-, and γ-tocopherol were obtained from Sigma Chemical (St. Louis, MO, USA). Finasteride and dutasteride were obtained from Wuhan W&Z Biotech (Wuhan, China). Agarose gel, Tris base, and 50X Tris/acetic acid/EDTA (TAE) were purchased from Bio-Rad Laboratories (Hercules, CA, USA). Foetal bovine serum (FBS; cat no. 16000044) and Roswell Park Memorial Institute medium (RPMI-1640; cat no. 31800022) were obtained from Gibco Life Technologies (Thermo Fisher Scientific, Waltham, MA, USA). Penicillin/streptomycin solution (100X) was purchased from Capricorn Scientific GmbH (Ebsdorfergrund, Germany). Ethanol, dimethyl sulphoxide (DMSO), acetic acid, trichloroacetic acid, and other chemical substances were obtained from RCI Labscan (Bangkok, Thailand). All other chemicals were of analytical grade.

### 2.2. Plant Material and Extraction

Rice bran of *Oryza sativa* Linn. cv. Tubtim Chumphae (RD69; TRB), Riceberry (RRB), and Mali Nil Surin (SRNC05053-6-2; MRB) was provided by Phrao Green Valley Co., Ltd. (Chiang Mai, Thailand). Rice bran of Yamuechaebia Morchor (YMCB 3 CMU; YRB) was obtained from Lanna Rice Research Center, Chiang Mai University, Thailand. Herbarium voucher specimens of TRB (PNPRDU63021), YRB (PNPRDU63022), RRB (PNPRDU63023), and MRB (PNPRDU63024) were deposited in the Pharmaceutical and Natural Products Research and Development Unit, Faculty of Pharmacy, Chiang Mai University. Rice bran (2 kg) was macerated in 95% (*v*/*v*) ethanol (ratio of solid/solvent: 1:3) for 24 h. The extract solutions were filtered through Whatman filter paper no. 4 and then no. 1 and concentrated by a vacuum evaporator (Hei-VAP value, Heidolph, Schwabach, Germany) at 50 °C. All extracts were stored in a sealed vial in the dark at 4 °C before further analysis.

### 2.3. Determination of Bioactive Compounds

#### 2.3.1. γ-Oryzanol and Tocopherols

The high-performance liquid chromatography (HPLC) conditions were adapted from a previous study [30]. The analytical system consisted of an Agilent 1220 Infinity DAD LC module (Agilent Technology, Palo Alto, CA, USA) and a fluorescent detector (Agilent 1260 FLD Spectra, Agilent Technology). The separation was performed on an Ultra C-18 column (250 mm × 4.6 mm, 5 µm particle size; Restek, Bellefonte, PA, USA). Briefly, 10 mg of the sample was diluted with 1 mL of isopropanol, mixed and then filtered through a 0.45 µm syringe filter into a 1.5 mL vial. Twenty microliter aliquots were injected. A mixture of acetonitrile/methanol/isopropanol in different ratios served as the mobile phase (solvent A: 50:40:10, *v*/*v*/*v*; solvent B: 30:65:5, *v*/*v*/*v*). The following procedure was used for the separation of both γ-oryzanol and tocopherols: isocratic elution with phase A for 5 min, followed by a 10 min linear gradient from phase A to 100% phase B, and a final 5 min isocratic elution with phase B. The column temperature was 25 °C with the flow at 1 mL/min. γ-Oryzanol was detected by a UV–VIS spectrophotometric detector at 325 nm. Tocopherols were detected by using a fluorescence detector with excitation and emission wavelengths at 290 and 330 nm, respectively. OpenLAB software (Agilent Technology) was used to acquire and process the data. The standard compounds were γ-oryzanol and the mixture of α-, β-, and γ-tocopherol.

#### 2.3.2. Total Phenolic Content

The total phenolic content (TPC) of all samples was determined using the Folin–Ciocalteu colourimetric method, as described in a previous study [31]. The calibration curve was plotted using the absorbance of standard gallic acid against its concentration in the range between 0.2 and 0.0016 mg/mL. The TPC was calculated according to the standard curve equation of gallic acid (*y* = 13.463*x* + 0.0406, *R*^2^ = 0.9991) and is expressed as mg gallic acid equivalents per 100 g dried sample (mg GAE/100 g). All the samples were prepared in triplicate.

#### 2.3.3. Total Flavonoid Content

An aluminium chloride colourimetric assay was used to estimate the total flavonoid content (TFC) of all extracts following the method described by Zeng et al. [32]. The calibration curve was created using different concentrations of EGCG (0.01–0.32 mg/mL) and its absorbance was measured at 515 nm. The standard curve equation of EGCG was y = 0.3587x + 0.0041 (*R*^2^ = 0.9993). The results are represented as mg EGCG equivalents per 100 g dried sample (mg EGCGE/100 g). All the samples were prepared in triplicate.

### 2.4. Cell Culture

DU-145 human prostate cancer cells were obtained from the American Type Culture Collection (Rockville, MD, USA). DU-145 was grown in RPMI-1640 containing 10% FBS and 1% antibiotics (100 μg/mL of streptomycin and 100 unit/mL of penicillin). DU-145 cells were maintained at 37 °C in a humidified incubator containing 5% CO_2_. The cells in passages 3–6 were used for all experiments.

### 2.5. Cell Viability Assay

The samples were tested to determine the non-cytotoxic concentration and cell viability of DU-145 cells by using SRB assay, as previously described [33]. Briefly, cells were seeded at a density of 1 × 10^4^ cells/well in a 96-well plate and incubated overnight for cell attachment in a 5% CO_2_ atmosphere at 37 °C. Cells were then exposed to five serial concentrations (0.0001–1 mg/mL) of the ethanolic rice bran extracts (TRB, YRB, RRB, and MRB), their bioactive compounds (ferulic acid, phytic acid, gallic acid, oleic acid, linoleic acid, and γ-linolenic acid; γ-oryzanol; and α-, β-, and, γ-tocopherol), and standard controls (dutasteride and finasteride). Control cells were treated with 10% (*v*/*v*) DMSO in incomplete RPMI-1640, whereas incomplete RPMI-1640 served as a blank. After 24 h treatment, the adherent cells were fixed with 50% (*w*/*v*) trichloroacetic acid for 30 min, and cells were washed with water and then air-dried. Cells were stained with 0.04% (*w*/*v*) SRB for 30 min. The unbound dye was removed by washing with 1% (*v*/*v*) acetic acid. The bound stain was solubilised with 10 mM Tris base, and absorbance was detected at 515 nm using a 96-well plate reader (EZ Read 400 Flexi, Biochrom, Cambridge, UK). The experiments were performed in triplicate. The highest non-toxic concentration that gave more than 80% cell viability was selected for further studies. The percentage of cell viability was calculated by Equation (1), where Abs denotes absorbance:

(1) $$\mathrm{Cell}\;\mathrm{viability}\;\left(\%\right)=\;\left(\frac{{\mathrm{Abs}}_{\mathrm{sample}}-{\mathrm{Abs}}_{\mathrm{blank}}}{{\mathrm{Abs}}_{\mathrm{control}}-{\mathrm{Abs}}_{\mathrm{blank}}}\right)\times 100.$$

### 2.6. RNA Extraction and Semiquantitative RT-PCR Analysis

#### 2.6.1. RNA Extraction

Total RNA was extracted from DU-145 cells treated with 0.10 mg/mL of the ethanolic rice bran extracts (TRB, YRB, RRB, and MRB), 0.01 mg/mL of bioactive compounds, 0.10 mg/mL of standard controls (finasteride and dutasteride), or untreated cells using the NucleoSpin^®^ RNA isolation kit (cat no. 740955.50; Macherey-Nagel, Duren, Germany) according to the manufacturer’s instructions. The concentration of isolated RNA was quantified using a Qubit 4 fluorometer (Invitrogen, Carlsbad, USA) and Qubit™ RNA HS Assay Kit (Invitrogen). The total RNA solution was kept at −20 °C until use.

#### 2.6.2. Semi-Quantitative RT-PCR

Complementary DNA (cDNA) was synthesised by using the RT-PCR Quick Master Mix (Toyobo, Osaka, Japan) according to the manufacturer’s instructions. Briefly, the 20 µL reaction mixture contained 1 µg of total RNA, 10 µL of the RT-PCR Quick Master Mix, 1 µL of manganese acetate, 1.2 µL of oligo dT primers (Integrated DNA Technologies, Coralville, IA, USA), and nuclease-free water. The transcript levels of the genes of interest (*SRD5A1*, *SRD5A2*, and *SRD5A3*) and the reference gene (glyceraldehyde 3-phosphate dehydrogenase (*GAPDH*)) were measured in triplicate. The sequences of primers are listed in Table 1. The cycle consisted of denaturation at 94 °C for 30 s, annealing at 50–55 °C for 30 s, and extension at 72 °C for 1 min; there were 40 amplification cycles.

The RT-PCR products were analysed by electrophoresis on 1% (*w*/*v*) agarose gels in a chamber with 1X TAE buffer at 100 V for 60 min. The gel was imaged with a Gel Doc™ EZ System (Version 3.0; Bio-Rad) to obtain images of the bands. Image Lab™ software (Bio-Rad) was used to analyse quantitatively the intensity of the bands. The expression of target genes was normalised by the GAPDH expression value and is expressed as the relative expression (*RE*) value. The percentage of SRD5A suppression was calculated according to Equation (2):

(2) $$SRD5A\;\mathrm{suppression}\;\left(\%\right)=\left(\frac{RE_{control}-\;RE_{sample}}{RE_{control}}\right)\times 100$$

### 2.7. Computational Method Details

#### 2.7.1. Protein and Ligand Preparation

**Figure 1:**
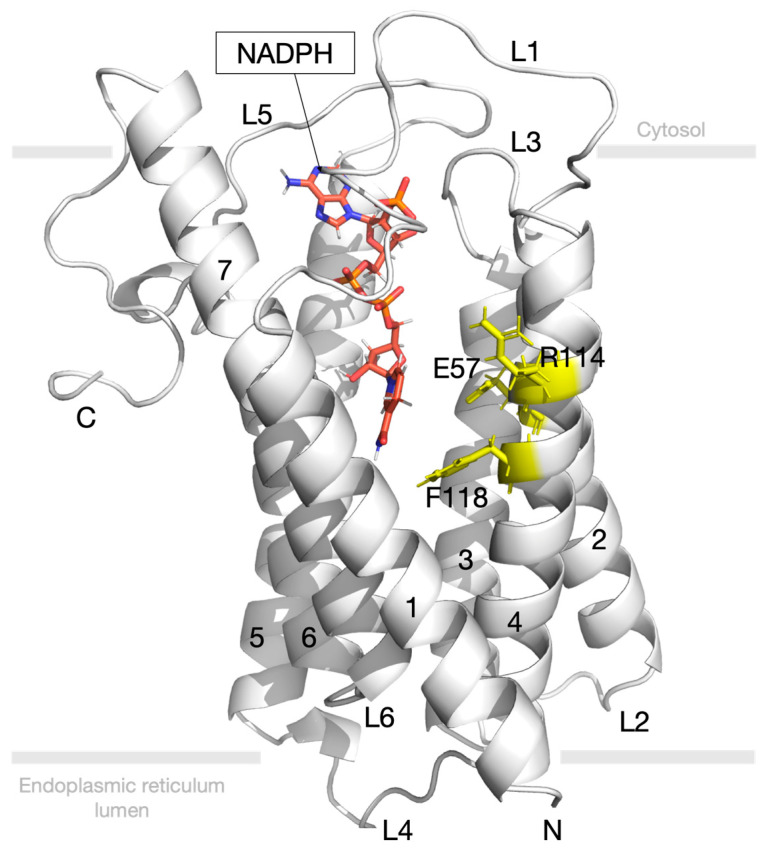
Structure of steroid human 5-alpha reductase 2 (SRD5A2) comprises seven transmembranes (7 TMs) and six loops. Dihydronicotinamide adenine dinucleotide phosphate (NADPH) (orange) is located inside the binding site. Crucial residues involving ligand interaction are coloured in yellow.

The structure of human SRD5A2 comprises seven transmembrane domains and six loops that construct a cavity inside SRD5A2 (Figure 1). A carboxyl-terminal side (C-terminal side) faces the cytosol, whereas an amino-terminal side (N-terminal) faces the endoplasmic reticulum lumen. The space between transmembrane (TM)1 and TM4 is the entry port that opens for the ligand to enter the binding site. Loop 1 (L1) has been suggested to be a gate domain that controls the NADPH/NADP^+^ exchange from the cytosol. A previous study reported that SRD5A2 catalyses the hydride transfer from NADPH to finasteride, resulting in the formation of a stable intermediate adduct, namely NADP–dihydrofinasteride (NADP–DHF), via a covalent bond [34]. Consequently, SRD5A2 is inhibited irreversibly. The key residues E57 (Glu57), R114 (Arg114), and F118 (Phe118) inside the SRD5A2 pocket have been suggested to interact with the intermediate adduct of finasteride and steroid substrate [34].

The crystal structure of human SRD5A2 in complex with NADPH and finasteride was retrieved from the Protein Data Bank (PDB) with the PDB ID 7BW1 [34]. SRD5A2 was prepared in Discovery Studio version 2.5 software. In the SRD5A2 binding pocket, finasteride is already fused with NADP^+^. The finasteride structure was removed by breaking the chemical bond, while the reduced form of NADP^+^ (NADPH) only remained in the substrate-binding cavity. SRD5A2 with NADPH in the binding pocket (Figure 1) is the state before ligand entry and formation of the intermediate adduct. This model was used to evaluate and compare the binding mode of ligands. The structure of finasteride retrieved from the crystal structure was used as a positive control.

**Figure 2:**
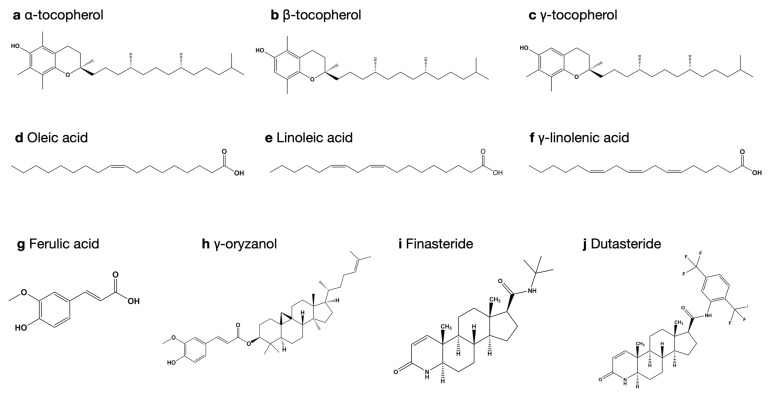
Chemical structure of selected ligands. (**a**) α-tocopherol, (**b**) β-tocopherol, (**c**) γ-tocopherol, (**d**) oleic acid, (**e**) linoleic acid, (**f**) γ-linolenic acid, (**g**) ferulic acid, (**h**) γ-oryzanol, (**i**) finasteride, and (**j**) dutasteride.

The three-dimensional structure of nine bioactive compounds (ferulic acid, phytic acid, oleic acid, linoleic acid, γ-linolenic acid, γ-oryzanol, and α-, β-, and γ-tocopherol), which can be purified from rice bran extracts and dutasteride, another positive control, were retrieved from the PubChem compound database and optimised with the Gaussian 09 program (Gaussian Inc., Wallingford, CT, USA) using the B3LYP model with a 6-31G (d, p) basis set [35]. Figure 2 illustrates the chemical structures of selected ligands.

#### 2.7.2. Molecular Docking Study for 5-Alpha Reductase 2

Nine compounds and two positive controls were docked into the binding site of SRD5A2 using AutoDock 4.2.6 and AutoDockTools, the graphical user interface [36]. In all dockings, a grid box was generated with a centre over the native finasteride position. Grid box dimensions were set to 34, 30, and 30 in x, y, and z dimensions, respectively. The grid spacing was kept at 0.375 Å. One hundred conformations were generated by Lamarckian genetic algorithm searches with an initial population size of 300 random positions and conformation. Each run had two stop criteria: a maximum of 2,500,000 energy evaluations and a maximum of 27,000 generations. The reference root-mean-square deviation (RMSD) was kept as the default, with a mutation rate of 0.02 and a crossover rate of 0.8. The RMSD tolerance of 2 Å was kept for clustering of docked poses and ranked according to their binding energy. The docked conformation with the lowest binding energy of the most populated cluster was selected.

#### 2.7.3. Molecular Dynamics Simulation

The best-docked complexes of native finasteride (positive control); α-, β-, and γ-tocopherol; the best top-three compounds; and SRD5A2 were selected for the MD simulation using the AMBER 14 program. The Amber ff14SB force field was used for the conformational analysis of protein systems [37,38]. For the non-standard unit in AMBER, antechamber was used to determine the GAFF atom type for structures of the four ligands and NADPH, and the restrained electrostatic potential (RESP) charges were employed for these ligands [39]. The systems were neutralised with 14 chloride counterions and centred in a 10 Å truncated octahedral box of pre-equilibrated TIP3P water molecules using the tLEaP program [40]. All MD simulations were carried out with the GPU-capable PMEMD.CUDA in AMBER14. The solvated structures were first energy-minimised to remove possible steric stress using the steepest descent (SD) method and the conjugate gradient techniques (CONJ) with a different part of the system [41]. They were then heated gradually from 0 to 310 K for 500 ps and equilibrated at 310 K at 1 atm pressure to obtain a stable density for 1000 ps. The unconstrained production simulations were run in an NPT ensemble at 310 K and 1 atm for 50 ns. The Langevin thermostat was used to maintain the temperature of the system [42], the SHAKE algorithm was used to constrain all of the chemical bond lengths involving hydrogen atoms [43], and the time step was set at 2 fs for all MD simulations.

#### 2.7.4. Trajectory Analysis

Visual molecular dynamics (VMD) [44] and PyMOL [45] were used to visualise and analyse MD trajectories. The structural analysis of the conformational ensemble was performed by evaluating the RMSD with the CPPTRAJ module implemented in AMBER 14 [46]. The binding free energy and decomposed binding free energy of SRD5A2/ligand complexes were evaluated by the molecular mechanics–generalised Born surface area (*MM*/*GBSA*) protocol using the MMPBSA.py module, as embedded in AMBER 14 [47,48]. The snapshots were extracted from 50 ns of MD trajectories for the analysis of the binding free energy; all water molecules and chloride counterions were removed prior to calculations. In *MM*/*GBSA*, binding free energy (∆*Gbind*) was estimated through Equation (3) [49]:

(3) $$\begin{array}{c} ∆G_{bind}\;=\;G_{complex}-G_{protein}-G_{ligand}\\ =∆H+∆G_{solvation}+T∆S\\ =∆E_{MM}+∆G_{GB}+∆G_{SA}-T∆S\\ =∆E_{vdw}+∆E_{ele}+∆G_{GB}+∆G_{SA}+T∆S \end{array}$$

where ∆*EMM* is the gas-phase interaction energy between protein and ligand, containing van der Waals interaction energy (∆*Evdw*) and electrostatic energy (∆*Eele*); ∆*GGB* and ∆*G* denote the polar and nonpolar desolvation free energy, respectively; and -*T*∆*S* indicates the conformational entropy contribution at temperature *T*, where *T* is the absolute temperature and *S* the entropy of the molecule. Here, the generalised Born (*GB*) approximation model was used to estimate the polar desolvation term (∆*GGB*) [50,51], while the solvent-accessible surface area (*SASA*) model with the LCPO model used to estimate the nonpolar desolvation term (∆*GSA*): ∆*GSA* = 0.0072 × ∆*SASA* [52].

### 2.8. Statistical Analysis

Data are expressed as the mean of three independent experiments ± standard deviation. Principal component analysis (PCA) of bioactive contents and *SRD5A* suppression was performed using a free trial version of XLSTAT (Addinsoft, New York, NY, USA). Linear correlation between variables is expressed by Pearson’s correlation coefficient (*r*). Analysis of variance (ANOVA) followed by post hoc analysis (Tukey’s test) was used to compare means and evaluate statistical differences. The null hypothesis was rejected at the calculated probability value of less than 5%. Both linear correlation and ANOVA were carried out in SPSS Statistics for Windows, Version 17.0 (SPSS Inc., Chicago, IL, USA).

## 3. Results and Discussion

### 3.1. Extraction Yield and Bioactive Compounds

The extraction yields, relative to 100 g of dried material, were 7.49% ± 0.89% (TRB), 5.68% ± 0.27% (YRB), 4.54% ± 0.92% (MRB), and 3.08% ± 1.67% (RRB); TRB had the highest yield among the four varieties of rice bran extracts. The physical appearances of all extracts were viscous semisolid and greasy with different colours due to pigment deposition in the pericarp or the bran of the rice kernel [26,53], specifically dark reddish-brown (TRB and YRB), black-purple (MRB), and dark purple (RRB).

The active constituents of rice extracts are presented in Table 2. Tocopherols are the major group of natural antioxidants in rice bran and have a similar chemical structure based on a 6-chromanol amphiphilic ring with one to three methyl groups and a phytyl tail with three chiral centres, resulting in α-, β-, γ-, and δ-tocopherol [26]. The highest content of α-tocopherol was found in TRB (20.76 ± 0.13 mg/kg extract), followed by YRB (12.52 ± 0.01 mg/kg extract), RRB (11.95 ± 0.04 mg/kg extract), and MRB (7.61 ± 0.01 mg/kg extract). Pigmented rice bran contains about 9.67–116.60 mg/kg of α-tocopherol, which is higher than that in non-pigmented rice bran [54]. β-Tocopherol is present at only minor concentrations in rice bran extracts [55], and the pair of β- and γ-tocopherol isomers is not completely separated using reversephase HPLC [30]. Hence, the pair of β- and γ-tocopherol was quantified and interpreted jointly as (β+γ)-tocopherol. The content of (β+γ)-tocopherol in YRB (50.98 ± 0.02 mg/kg extract) was much higher than in the other rice bran extracts, while δ-tocopherol was not detected in the extracts. These results are in agreement with several studies, where α- and β-tocopherol are predominantly present in rice bran and rice whole grain [55,56].

Rice bran is a major source of γ-oryzanol, a mixture of steryl ferulates, and speculated to be the primary constituent of rice bran oil [26]. The content of γ-oryzanol was enriched in TRB (8600.45 ± 0.13 mg/kg extract) and RRB (9174.01 ± 0.09 mg/kg extract). It has been reported that the mean value of γ-oryzanol in non-pigmented rice bran is approximately 3067.10 mg/kg [54]. Overall, pigmented TRB and RRB contained a high content of γ-oryzanol, and black-purple rice (MRB) had the lowest amount of tocopherols and γ-oryzanol. It has been suggested that environmental factors as well as the origin and genotype of rice varieties influence the composition of steryl ferulates, resulting in the variation of the γ-oryzanol profiles in black-purple rice varieties [57].

Phenolic and flavonoid contents were measured by colourimetric methods. Highest levels of TPC and TFC were found in red rice bran (YRB): 254.97 ± 5.20 mg GAE/100 g dried sample and 880.16 ± 22.86 mg EGCGE/100 g dried sample, respectively. Phenolic acids can be classified as free, conjugated, and bound. The free form is suggested to be easily extracted from rice bran [53]. Phenolic compounds, including phenolic acids and flavonoids, are mainly present in pigmented rice and act as metal ion chelators, free radical scavengers, and reducing agents [53,58]. The foremost phenolic acids found in bran include ferulic acid (56–77% of total phenolic acids), followed by *p*-coumaric acid, sinapic acid, gallic acid, protocatechuic acid, *p*-hydroxybenzoic acid, vanillic acid, and syringic acid [46]. A previous study reported that Thai red rice bran contains a higher TPC than black and white rice bran extracts, resulting in better antioxidant activity [59]. The main flavonoids in non-pigmented rice varieties are flavones, whereas proanthocyanidins and anthocyanins are primarily found in pigmented rice varieties [54,60]. Anthocyanins and proanthocyanidins are responsible for purple-to-blue pigmentation and red pigmentation, respectively [26,61]. Moreover, a study reported that red and purple bran rice has greater TPC and TFC than light-coloured bran rice and other cereals due to higher concentrations of proanthocyanidins and anthocyanins, respectively [62].

Several studies have reported the use of topical α-tocopherol in cosmetic applications and treatments of cutaneous diseases such as wounds, sunburn, atopic dermatitis, and hair loss [63,64,65]. A recent study reported that α-tocopherol-loaded hydrogel promoted the healing of the dorsal skin injury in a rat model. Based on the histopathological results, the α-tocopherol-treated group showed epidermal proliferation and the generation of new hair follicles [63]. In addition, α-tocopherol and α-tocopheryl acetate lotions showed the acceleration of the hair growth rate in a rabbit model within two weeks [66]. Nevertheless, whether tocopherols affect an androgen-dependent pathway involving AGA has not been directly observed. Therefore, tocopherols were selected for further study to determine their effect on *SRD5A* isozyme expression and for a molecular docking study. In addition, other bioactive compounds in rice bran extracts, such as ferulic acid [67], phytic acid [68], oleic acid [27,69], linoleic acid [27,69,70], γ-linolenic acid [69,71], and γ-oryzanol [28], which are involved in androgen metabolism pathways, were compared to tocopherols and standard controls (finasteride and dutasteride).

### 3.2. Effect on the Expression of 5-Alpha Reductase Isoenzymes

The activity of SRD5A in hair follicles was first identified by Takayasu et al. [72]. *SRD5A1* and *SRD5A2* gene expression levels in men were about threefold higher than in women, resulting in the higher prevalence of AGA among men compared with women [4]. The expression of *SRD5A3* has been suggested to be a predisposing factor to AGA development [18,19,20]. Moreover, the absence of temporal regression and baldness in cases of *SRD5A* deficiency supports the crucial role of *SRD5A* in the pathogenesis of AGA [73]. Downregulation of *SRD5A* gene expression could lead to a reduction in their protein translation in the downstream pathways involving the pathology of AGA. Several studies have indicated that the DU-145 human androgen-insensitive prostate adenocarcinoma cell line expresses the three types of *SRD5A*; these cells have been used in the present study to observe the regulation of *SRD5A* gene expression [16,28,74,75]. With regards to these, the effects of the selected bioactive compounds and rice bran extracts on *SRD5A1*, *SRD5A2*, and *SRD5A3* expression were investigated. The percentage of *SRD5A* suppression and the relative expression of each *SRD5A* gene are given in Supplementary Materials (Table S1 and Figure S1).

**Figure 3:**
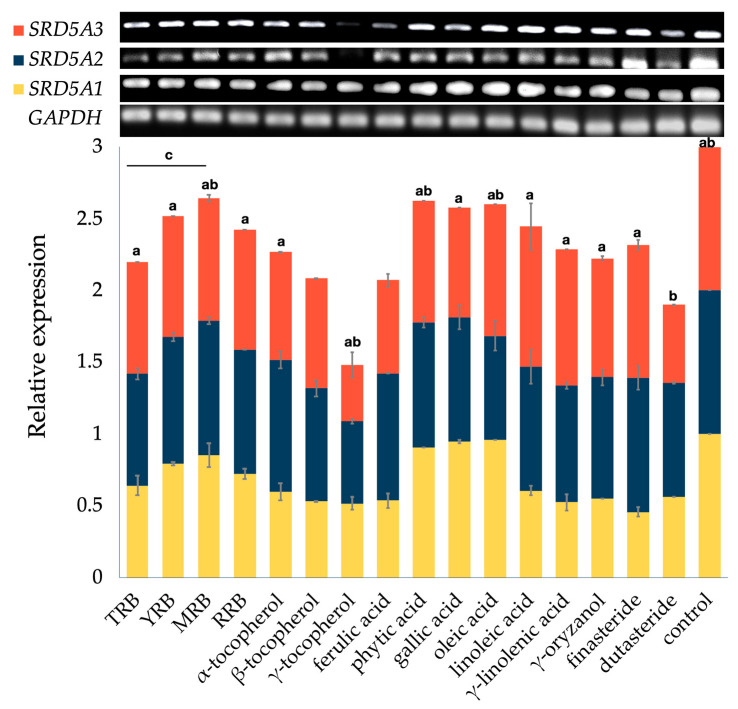
Effects of selected bioactive compounds and rice bran extracts on 5α-reductase isoenzyme (*SRD5A*) expression in DU-145 cells treated with 0.10 mg/mL of ethanolic rice bran extracts (TRB, YRB, RRB, and MRB), 0.01 mg/mL of selected bioactive compounds, and 0.10 mg/mL of standard controls (finasteride and dutasteride). A statistical significance in comparison to dutasteride and finasteride is indicated as a and b (*p* < 0.05), respectively. A significant difference between TRB and other extracts is expressed as c (*p* < 0.05).

The four rice bran extracts significantly decreased the mRNA expression levels of *SRD5A1*, *SRD5A2*, and *SRD5A3* compared with the negative control groups in the following order: TRB > RRB > YRB > MMB (Figure 3). Interestingly, treatment with TRB greatly decreased the expression levels of *SRD5A1*, *SRD5A2*, and *SRD5A3* by 35.79% ± 6.94%, 22.26% ± 3.73%, and 21.97% ± 0.01%, respectively (Table S1). Finasteride, a dual inhibitor of SRD5A2 and SRD5A3, suppressed *SRD5A1*, *SRD5A2*, and *SRD5A3* by 54.21% ± 3.05%, 6.48% ± 8.22%, and 7.70% ± 3.58%, respectively. However, there were no significant differences between TRB and finasteride regarding *SRD5A* gene suppression. Dutasteride, a triple inhibitor of SRD5As, downregulated *SRD5A1*, *SRD5A2*, and *SRD5A3* by 43.50% ± 0.01%, 20.84% ± 0.54%, and 45.57% ± 0.03%, respectively (Table S1). Among the selected bioactive compounds, the mRNA levels of *SRD5A1*, *SRD5A2*, and *SRD5A3* were downregulated markedly by γ-tocopherol (0.10 mg/mL), specifically 48.32% ± 4.29%, 42.57% ± 1.91%, and 61.04% ± 9.10%, respectively (Table S1). The overall effect of γ-tocopherol on *SRD5A* gene suppression was significantly greater than dutasteride. However, complete downregulation of *SRD5A* genes may not be the best choice for AGA patients, considering the required androgen balance for normal health and tissue homeostasis [3,4]. In skin, androgens also regulate sebum production and secretion, wound healing, cutaneous barrier formation, and hair growth [4]. Undesirable side effects of long-term use of SRD5A inhibitors on skin changes have been reported, including dry skin, thinning skin, changes in skin texture and tone, and penile and scrotal shrinkage [76,77]. Moreover, excessive suppression may lead to ejaculation problems, erectile dysfunction, sexual anhedonia, decreased sperm count, gynaecomastia, and loss of libido in patients [78].

The *SRD5A1* mRNA level was significantly lower after treatment with most of the bioactive compounds, including tocopherols, ferulic acid, linoleic acid, γ-linolenic acid, γ-oryzanol, and standard control groups (finasteride and dutasteride), compared with the negative control group (Figure S1a). This result is similar to previous studies [28,79]. The extracts (0.50 mg/mL), including *Nelumbo nucifera*, *Sesamum indicum*, and bran of *O. sativa*, greatly diminished *SRD5A1* expression, suggesting that a high content of linoleic acid is responsible for the activity with a synergist of ferulic acid, vanillic acid, phytic acid, and γ-oryzanol [79]. Nevertheless, in this study, the *SRD5A1* mRNA level slightly decreased in the groups treated with phytic acid, gallic acid, and linoleic acid. The concentration of standard bioactive compounds (0.01 mg/mL) and rice bran extracts (0.10 mg/mL) used in this study was lower than the concentration used in the previous study, suggesting that the *SRD5A* genes may be suppressed in a concentration-dependent manner.

The expression of *SRD5A2* was not remarkably changed with the treatments of YRB, MRB, RRB, and the major compounds (Figure S1b). *SRD5A2* was only downregulated in the cells treated with TRB, β- and γ-tocopherol, oleic acid, γ-linolenic acid, and dutasteride. The *SRD5A2* suppression exerted by the four extracts was in the following order: TRB > RRB > YRB > MMB. TRB suppressed *SRD5A2* by 22.26% ± 3.73%, which was not significantly different compared with standard dutasteride (20.84% ± 0.54%). This suppression might be from the synergistic effect of β- and γ-tocopherol, oleic acid, and γ-linolenic acid in the extract. The *SRD5A3* mRNA level was downregulated significantly in cells treated with all forms of tocopherol, phytic acid, phenolic acids, and γ-oryzanol compared with the control group, but it was not altered in groups treated with unsaturated fatty acids or finasteride (Figure S1c).

The sum of overall bioactive compounds (α-, β- and γ-tocopherol; γ-oryzanol; TPC; and TFC) was ranked in descending order: TRB > RRB > YRB > MMB. In addition, the overall results of *SRD5A* mRNA expression levels from all four rice bran extracts were arranged in the order of decreasing suppression: TRB > RRB > YRB > MMB. Taken together, these bioactive compounds provided potential synergy that enhances the downregulation of *SRD5A* genes.

### 3.3. Correlation Analysis

#### 3.3.1. Pearson’s Correlation

Pearson’s correlation coefficients (*r*) between the potential suppressive effect on *SRD5A* expression and the content of bioactive compounds in four rice bran extracts (α-tocopherol, (β+γ)-tocopherol, γ-oryzanol, TPC, and TFC) were determined to estimate the relationship between variables. The correlation coefficients are classified into four levels: particularly high (*r* > 0.9), high (0.9 > *r* > 0.7), moderate (0.7 > *r* > 0.5), and poor (*r* < 0.5) [80]. There were strong and significant linear relationships between the content of α-tocopherol and the suppressive effect on *SRD5A1* (*r* = 0.814, *p* < 0.01), *SRD5A2* (*r* = 0.917, *p* < 0.01), and *SRD5A3* (*r* = 0.943, *p* < 0.01). In contrast, a previous study reported a significant positive linear correlation between *SRD5A1* suppression and the unsaturated fatty acid content (*r* = 1.00, *p* < 0.01) and the linoleic acid content (*r* = 1.00, *p* < 0.01) [28]. Because the rice bran extracts in the previous study were obtained by supercritical CO_2_ extraction, the bioactive compounds in extracts were different from our study, especially the unsaturated fatty acid content [28].

Regarding HPLC results, YRB contained the highest content of (β+γ)-tocopherol among rice bran extracts, followed by TRB, RRB, and MRB. Furthermore, treatment with γ-tocopherol exhibited the greatest suppression of *SRD5A1*, *SRD5A2*, and *SRD5A3*. However, there was not a significant relationship between the content of (β+γ)-tocopherol in rice bran extracts and their suppression of *SRD5A* mRNA levels. Instead, there was a strong relationship between the α-tocopherol content and the suppressive effects on these genes. The most abundant form of tocopherols in pigmented rice bran was α-tocopherol, followed by γ-, β-, and δ-tocopherol [54]. In addition, a study reported that α-tocopherol is the major tocol in two Taiwanese rice varieties [26]. TRB showed the highest α-tocopherol content, followed by YRB, RRB, and MRB. There was a clear concordance between α-tocopherol and the suppressive effects on *SRD5A* genes. The content of γ-tocopherol in YRB may be lower than the content of α-tocopherol, resulting in the minor effect on gene regulation. These findings indicate that the expression levels of all *SRD5A* genes are greatly downregulated by TRB, suggesting that the higher α-tocopherol content contributes to the more pronounced effect.

#### 3.3.2. Correlation by Principal Component Analysis

**Figure 4:**
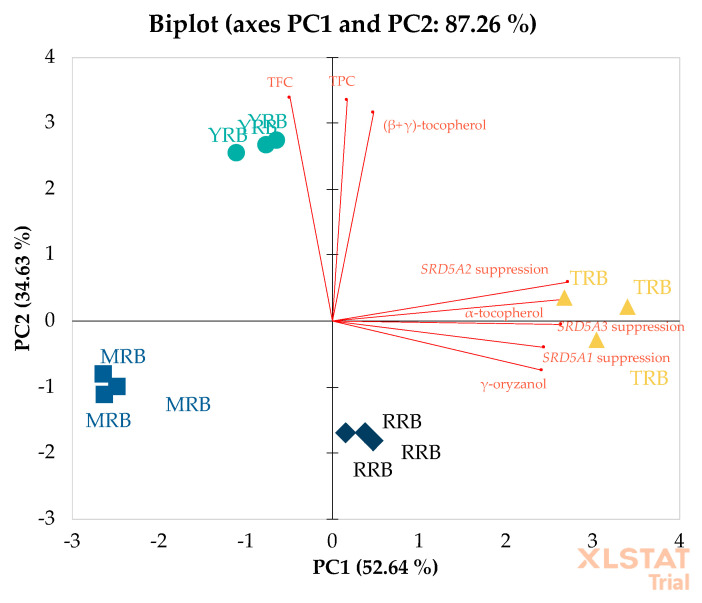
Principal component analysis (PCA) biplot of rice bran extracts and their biological and phytochemical properties. Rice bran extracts of Tubtim Chumphae (TRB), Yamuechaebia Morchor (YRB), Mali Nil Surin (MRB), and Riceberry (RRB). Total phenolic content (TPC). Total flavonoid content (TFC).

PCA was performed to classify the rice bran extracts and cluster the samples. Correlation between rice bran extracts (TRB, YRB, MRB, and RRB) and their biological activity (*SRD5A* suppression) and biological contents (α-tocopherol, (β+γ)-tocopherol, γ-oryzanol, TPC, and TFC) is shown as a PCA biplot in Figure 4. The PCA space distributed 52.64% in PC1 and 34.63% in PC2. The data were separated into four clusters of rice extracts across the PCA space. YRB was separated from other extracts due to the TPC, the TFC, and the content of (β+γ)-tocopherol. TRB showed a high correlation between *SRD5A* gene suppression and the contents of α-tocopherol and γ-oryzanol. RRB was slightly correlated with the content of γ-oryzanol, whereas MRB was not correlated with any variables. Regarding the groups and clustering seen in PCA, the biological activities and biological contents of samples were speculated to be similar. However, there was a strong correlation between the suppression of *SRD5A* genes and the content of α-tocopherol in TRB.

### 3.4. Molecular Docking Study for 5-Alpha Reductase 2

Both *SRD5A1* and *SRD5A2* are expressed and active in scalp hair follicles. Specialised fibroblasts in hair follicles or DPC, known as androgenic targets, induce surrounding epidermal cells to form hair follicles and regulate the hair growth cycle [81]. *SRD5A2* is expressed mainly in DPC obtained from scalp hairs, and its activity is 14-fold higher than in the remaining hair follicles [4]. In addition, the absence of AGA in males with congenital SRD5A2 deficiency provides strong evidence that SRD5A2 activity is the most importance factor in AGA development [82]. Even though it is more favourable for SRD5A1 to catalyse androstenedione as a substrate to generate 5α-androstenedione, SRD5A2 has been implicated mostly in the reduction of T to DHT [6,83,84]. Furthermore, finasteride, a selective SRD5A2 inhibitor, is a Food and Drug Administration (FDA)-approved drug to treat AGA and has proven favourable efficacy in increasing hair density and hair diameter [85]. Thus, SRD5A2 was selected to perform the further molecular docking with selected bioactive compounds of rice bran extracts.

Molecular docking was performed to predict the potential target/ligand interaction. The binding free energy, which indicates the ligand-binding possibilities with target protein SRD5A2, was calculated. The predicted binding free energies of finasteride, dutasteride, and nine bioactive compounds are ranked and presented in Table 3. The ranking is based on the binding affinity of the SRD5A2/ligand complex; the lowest binding energy (highest negative value) is projected to specify the best-possible interaction. The results indicated that the binding energy of finasteride (−10.13 kcal/mol) was lower than that of dutasteride (−8.75 kcal/mol). These results are in agreement with a previous study that indicated finasteride more specifically inhibits SRD5A2 (IC_50_ = 14.3 ± 2.7 nM) compared with dutasteride (IC_50_ = 57.0 ± 6.8 nM) [5,16]. Several studies have shown that both synthetic and plant-derived unsaturated fatty acids inhibit SRD5A and, consequently, block the conversion of T to DHT [27,33,86,87]. Ferulic acid, γ-oryzanol, and phytic acid appeared to have unfavourable interactions. Similarly to a previous study, there was no correlation between SRD5A inhibitory activity and the TPC [88]. Strikingly, tocopherols displayed a higher affinity towards SRD5A2 compared with dutasteride and other active constituents. The binding free energies of tocopherol are in ascending order: β-tocopherol (−9.83 kcal/mol), γ-tocopherol (−9.53 kcal/mol), and α-tocopherol (−9.47 kcal/mol). Among all considered compounds, finasteride and tocopherols were subjected to MD simulation.

### 3.5. Molecular Dynamics Simulation

#### 3.5.1. Stability of the Molecular Dynamics Trajectories

**Figure 5:**
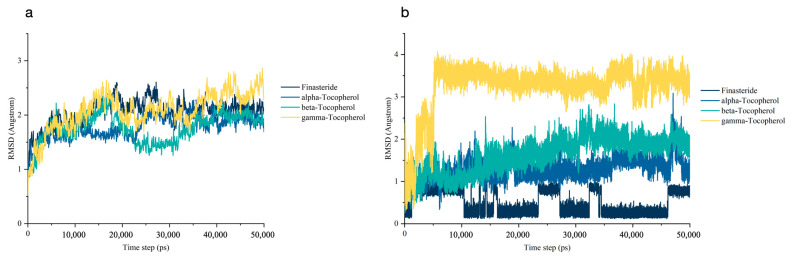
Root-mean-square deviation (RMSD) analysis over 50 ns MD stimulation time: (**a**) RMSD plot of the steroid 5α-reductase 2 (SRD5A2) in complex with each ligand (finasteride and α-, β-, and γ-tocopherol). (**b**) RMSD plot of each ligand.

The structural dynamics at the atomistic level of SRD5A2 upon interacting with all ligands were carried out by MD trajectories over a 50 ns simulation time. The RMSD values of the protein backbone atoms were calculated to determine the stability of each SRD5A2/ligand complex and were plotted relative to the first frame of the original structure (Figure 5a). The RMSD values of the SRD5A2 backbone for all ligands were in the range of 2 Å, establishing their overall stability over the explored timescale [89]. There were large fluctuations of SRD5A2/α-tocopherol and SRD5A2/β-tocopherol at 10–20 ns and 20–30 ns, respectively. All systems reached the same state after 40 ns.

In the case of the ligand RMSD (Figure 5b), finasteride was stable throughout the 50 ns MD simulation. RMSD plots of α- and β-tocopherol showed almost the same pattern. The RMSD values of three ligands (finasteride and α- and β-tocopherol) were less than ∼2 Å and remained stable to the end of the 50 ns simulation, indicating that these ligands have a stable conformation in the binding site. However, the RMSD of γ-tocopherol increased from the beginning of the MD simulation until approximately 5 ns. Hence, γ-tocopherol dramatically underwent a conformational change at the starting period and then maintained a constant trend until 50 ns. Overall, finasteride and α- and β-tocopherol exhibited a reciprocal stabilisation in the SRD5A2 pocket, contributing to an increase in the binding affinity of the ligands.

#### 3.5.2. Binding Free Energy Analysis

The binding free energy of all ligands towards SRD5A2 was estimated using the snapshots obtained from a 50 ns MD simulation [90]. The different individual energies including van der Waals forces (VDW), electrostatic energy (EEL), nonpolar contribution to the solvation free energy (ESURF), ∆G_gas_, and ∆G_solv_ are summarised into the total binding free energy, or ∆G_Total_ (Table 4). α-Tocopherol possessed the highest binding affinity against SRD5A2, with ∆G_Total_ of −54.55 ± 3.67 kcal/mol. VDW, a major support of ∆Ggas, was more favourable in all ligands compared with electrostatic interaction.

#### 3.5.3. Decomposition of Binding Free Energy

**Figure 6:**
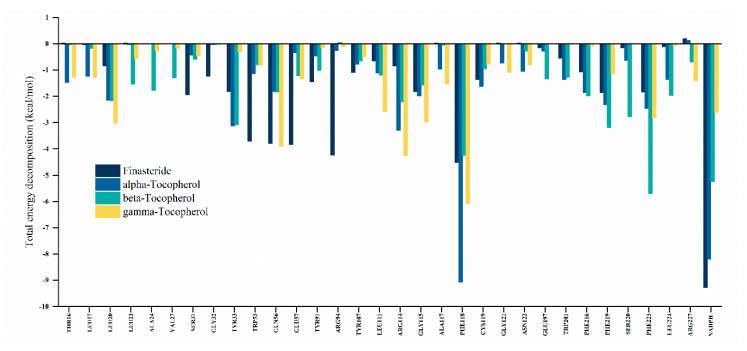
Energy decomposition of amino acid residues in the binding site towards the ligands (finasteride and α-, β-, and γ-tocopherol).

The free energy contribution of each amino acid residue of the SRD5A2 pocket for ligand interaction was elucidated by energy decomposition analysis using MM/GBSA with the configurations based on a 50 ns MD simulation. A residue is considered a favourable contributor to ligand binding when the total energy decomposition is more negative than −1 kcal/mol [71]. All hotspot residues and NADPH as a donor cofactor are represented in Figure 6. The most crucial residue in all SRD5A2/ligand complexes is Phe118, which contributed favourably to the ligand binding in the following order: α-tocopherol (−9 kcal/mol), β-tocopherol (−6 kcal/mol), finasteride (−4.5 kcal/mol), and γ-tocopherol (−4.2 kcal/mol). It is obvious that finasteride was more likely to interact with NADPH via VDW and *pi*-alkyl interaction. Nevertheless, a covalent bond has been suggested to connect the nicotinamide C-4 atom of NADPH and the C-2 atom at the pyridone ring of finasteride and then create an intermediate adduct between NADPH and finasteride (NADP–dihydrofinasteride) [34,91]. In addition, the key residues in the SRD5A2 binding cavity, such as Ser31, Gly32, Trp53, Glu57, Try91, and Arg94, were prone to form interactions with finasteride. Conversely, tocopherols were mainly involved with the hotspot residues Lue20, Leu11, Arg114, Gly115, Phe219, and Phe233. These findings indicate that finasteride and tocopherols interact with crucial residues in the SRD5A2 pocket at different levels and with distinct binding patterns.

### 3.6. Post-Molecular Dynamics Simulation Binding Mode Analysis

The dynamic data at atomic spatial resolution revealed that α-tocopherol, which can be found in rice bran extract, is considered a promising SRD5A2 inhibitor. Our preliminary molecular docking study indicated that α-tocopherol, with a binding free energy of −9.47 kcal/mol, had less interaction with SRD5A2 compared with other tocopherols and finasteride. According to the stability of trajectories, all systems of the SRD5A2/ligand complex reached the same state after 40 ns (Figure 5a). The ligand RMSD values of α- and β-tocopherol were quite low, within the acceptable limit of less than 2 Å, whereas the RMSD value of γ-tocopherol was higher (Figure 5b). These data suggest that α- and β-tocopherol undergo a slightly conformational change. However, the binding free energy analysis indicates that α-tocopherol possesses the most favourable interaction by the energy of −54.55 kcal/mol.

**Figure 7:**
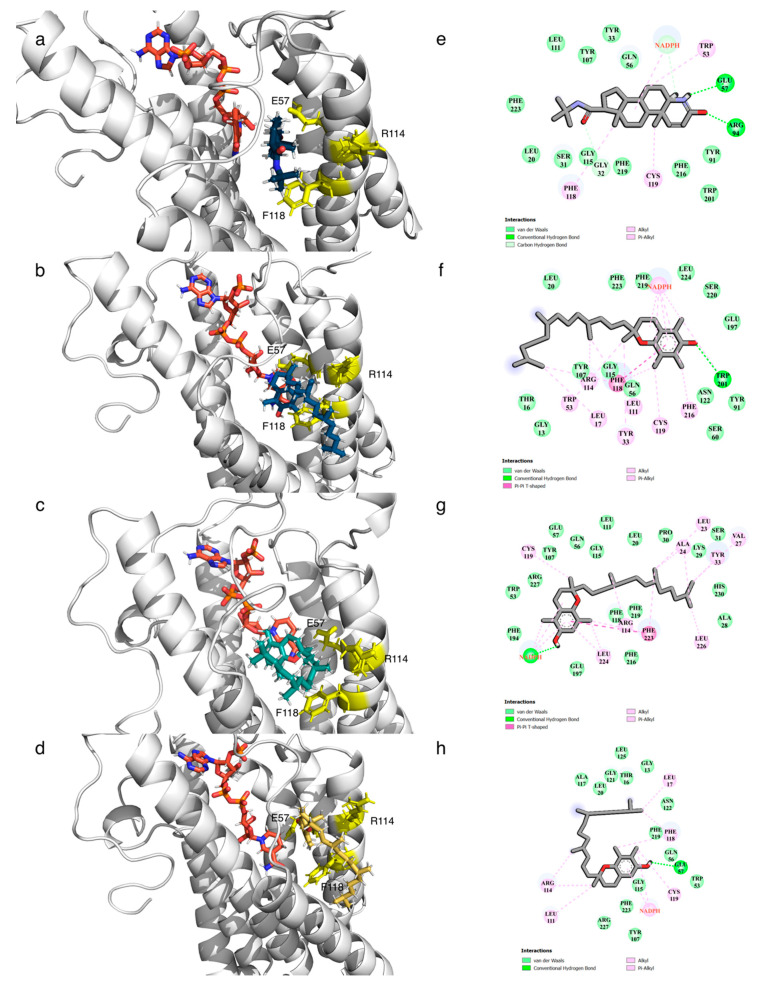
Molecular interaction patterns of 3D and 2D interactions of the ligands (finasteride and α-, β-, and γ-tocopherol) in the pocket of 5α-reductase type 2 after molecular dynamics simulation; the interacting residues are highlighted with different colours: (**a**,**e**) binding pose of finasteride, (**b**,**f**) binding pose of α-tochopherol, (**c**,**g**) binding pose of β-tocopherol, and (**d**,**h**) binding pose of γ-tocopherol.

Molecular interaction and binding mode conformations of key residues were executed using the final snapshot from the MD simulation of each complex and constructed into 3D and 2D plots (Figure 7). The study demonstrated that tocopherols occupy the SRD5A2 binding pocket similarly to finasteride but display distinct binding patterns. SRD5A2 contains seven transmembrane α-helices with NADPH buried in cytosolic loops [7]. The post-MD binding pose showed that all ligands enter the port and mainly interact with residues in TM2 and TM4.

α-Tocopherol formed different intermolecular interactions between its benzene ring and residues in the binding pocket, including Phe118 (*pi-pi* interaction) and Trp201 (hydrogen bonding). The energy decomposition analysis indicates that NADPH is likely to interact with a chromanol head of *α*-tocopherol via hydrophobic interactions. The binding environment and the formation of an intermediate adduct between NADPH and α-tocopherol may provide better interaction inside the binding pocket. Moreover, the total energy decomposition of the residue Phe118 is the most negative in the SRD5A2/α–tocopherol complex (−9 kcal/mol). A previous study reported that a substitution mutation at the residue Phe118 in the SRD5A2 binding cavity could dramatically restrain the binding of testosterone [92]. Together with our findings, the residue Phe118 is the most crucial amino acid that is prone to interact with steroid substrates and ligands, including α-tocopherol.

By contrast, finasteride had strong hydrogen bond interaction with residues Arg94 and Glu57. The residue Phe118 interacted with finasteride via hydrophobic interaction (−4.5 kcal/mol) but less compared to its interaction with α-tocopherol. In accordance with our results, a previous study suggested that residues Glu57 and Arg114 interact with an intermediate adduct of finasteride and NADPH in the binding pocket [34]. In addition, residues Tyr235, Asp241, and Lys244 interact with finasteride inside the SRD5A2 binding cavity [91], but these residues were not significantly observed in this study.

Although β- and γ-tocopherol form a number of notable interactions with residues in the SRD5A2 binding pocket, the complexes were much less stable than α-tocopherol and finasteride. This may be due to fact that the chromanol ring of α-tocopherol contains three methyl groups at the C5-, C7-, and C8-positions, while β- and γ-tocopherol have two methyl groups. Consequently, the residues in the pocket are likely to form a hydrophobic interaction with these methyl groups of α-tocopherol, resulting in the stabilisation of ligands [93]. The rigid moieties at the phytyl tail of β- and γ-tocopherol might contribute to the steric hindrance effect and affect the binding pocket insertion [94].

We performed MD simulations to screen for promising SRD5A2 inhibitors and gain insight into the stability and overall dynamics of each ligand in the SRD5A2 pocket before the intermediate adduct is formed. However, the present study only provides a proof-of-concept by focusing on the molecular interactions of the ligand in the binding site without a membrane environment. Because SRD5A2 is a membrane-embedded steroid reductase, the influence of the membrane environment is an important factor for the protein conformational dynamics [95]. A previous advanced MD study has demonstrated that steroid substrates presumably access the SRD5A2 binding pocket from the lipid bilayer through the opening between TM1 and TM4. Furthermore, high conformation dynamics of the cytosolic region were observed during the NADP+/NADPH exchange [34]. Membrane–protein interactions may affect ligand entry, ligand binding modes, and SRD5A2 structural conformations. Thus, future MD studies of SRD5A2 should include the lipid membrane to evaluate its influence on the system. The experimental study of promising ligands towards SRD5A2 needs further investigation to confirm the inhibitory activity.

## 4. Conclusions

Ethanolic TRB rice bran extract, a dark-brown semisolid extract, contained a decent amount of α-tocopherol and had the largest sum of overall bioactive compounds (γ-oryzanol; α-, β-, and γ-tocopherol; TPC and TFC). TRB also showed the highest antiandrogenic suppression of *SRD5A1*, *SRD5A2*, and *SRD5A3* mRNA. There was no statistically significant difference in the suppression of the mRNA expression of the three types of *SRD5A* between TRB and finasteride (FDA-approved for anti-hair loss treatment). The linear relationship and PCA indicated that the content of α-tocopherol shows a strong contribution to the suppression of all *SRD5A* genes. Strong evidence from previous studies indicates that SRD5A2 activity is implicated mostly in the reduction of T to DHT and expressed mainly in DPC obtained from hair follicles, highlighting the vital role of SRD5A2 in AGA development. SRD5A2 was subsequently docked with selected bioactive compounds based on the prior results of the studies indicating the involvement in androgen pathways. MD simulation demonstrated that α-tocopherol forms a stable interaction with SRD5A2 similarly to finasteride; it forms hydrophobic and hydrogen bond interactions with crucial amino acid residues in the SRD5A2 binding pocket, supporting the potential inhibition of this bioactive compound in TRB. Our findings bring new insights that TRB suppresses *SRD5A* gene expression as well as SRD5A2 activity. However, additional studies of enzymatic inhibition need to be evaluated. This will help to progress the uses of this rice by-product for future pharmaceutical and cosmeceutical applications as anti-hair loss products.

## Figures and Tables

**Table 1:** The sequences of the primers used for RT-PCR.

Primer	NCBI Reference Sequence	Forward Primer	Reverse Primer
*SRD5A1*	001047.4	AGCCATTGTGCAGTGTATGC	AGCCTCCCCTTGGTATTTTG
*SRD5A2*	000348.4	TGAATACCCTGATGGGTGG	CAAGCCACCTTGTGGAATC
*SRD5A3*	024592.5	TCCTTCTTTGCCCAAACATC	CTGATGCTCTCCCTTTACGC
*GAPDH*	001289745.3	GGAAGGTGAAGGTCGGAGTC	CTCAGCCTTGACGGTGCCATG

**Table 2:** Bioactive compounds of four types of rice bran extracts.

Sample	α-Tocopherol	(β+γ)-Tocopherol	γ-Oryzanol	TPC	TFC
	mg/kg Extract	mg/kg Extract	mg/kg Extract	mg GAE/100 g	mg EGCGE/100 g
TRB	20.76 ± 0.13	23.32 ± 0.01	8600.45 ± 0.13	180.44 ± 6.42	569.01 ± 90.42
YRB	12.52 ± 0.01	50.98 ± 0.02	3773.17 ± 0.01	254.97 ± 5.20	880.16 ± 22.86
MRB	7.61 ± 0.01	3.51 ± 0.01	ND	125.67 ± 0.49	545.30 ± 48.33
RRB	11.95 ± 0.04	16.97 ± 0.01	9174.01 ± 0.09	56.47 ± 2.82	441.49 ± 12.39

Rice bran extracts of Tubtim Chumphae (TRB), Yamuechaebia Morchor (YRB), Mali Nil Surin (MRB), and Riceberry (RRB). Total phenolic content (TPC) presented as mg gallic acid equivalents per 100 g of dried sample (mg GAE/100 g). Total flavonoid content (TFC) expressed as mg (−)-epigallocatechin gallate equivalents per 100 g of dried sample (mg EGCGE/100 g). Not detected (ND).

**Table 3:** Binding free energy of finasteride, dutasteride, and nine bioactive compounds with 5α-reductase 2.

Compounds	AutoDock Binding Free Energy, ∆G (kcal/mol)
Native finasteride	−10.13
β-Tocopherol	−9.83
γ-Tocopherol	−9.53
α-Tocopherol	−9.47
Dutasteride	−8.75
γ-Linolenic acid	−7.03
Linoleic acid	−6.62
Oleic acid	−6.49
Ferulic acid	−5.22
γ-Oryzanol	−4.26
Phytic acid	−2.02

**Table 4:** Estimated binding free energy of the complex of 5α-reductase 2 with the ligands (finasteride and α-, β-, and γ-tocopherol) using molecular mechanics–generalised Born surface area (MM/GBSA).

Compounds	Binding Free Energy (kcal/mol)						
	VDW	EEL	EGB	ESURF	∆G_gas_	∆G_solv_	∆G_Total_
Finasteride	−53.93 ± 3.23	−18.81 ± 3.41	32.36 ± 3.03	−6.15 ± 0.24	−72.74 ± 4.33	26.20 ± 2.95	−46.54 ± 3.12
α-Tocopherol	−64.97 ± 3.21	−2.26 ± 1.68	20.10 ± 1.80	−7.42 ± 0.39	−67.23 ± 3.68	12.68 ± 1.77	−54.55 ± 3.67
β-Tocopherol	−60.01 ± 4.30	−8.13 ± 2.90	25.88 ± 2.35	−8.15 ± 0.55	−68.14 ± 5.02	17.73 ± 2.10	−50.40 ± 4.33
γ-Tocopherol	−51.24 ± 4.41	−5.71 ± 4.69	23.02 ± 3.76	−6.77 ± 0.47	−56.96 ± 5.54	16.25 ± 3.65	−40.71 ± 3.88

The energy of each compound comprised individual energy terms, including van der Waals forces (VDW), electrostatic energy (EEL), the electrostatic contribution to the solvation free energy (EGB), and nonpolar contribution to the solvation free energy (ESURF). The binding free energy terms are given by ∆G_gas_ (gas-phase free energy) = VDW + EEL, ∆G_solv_ (solvation free energy) = EGB + ESRUF, and ∆G_Total_ (total binding free energy) = ∆G_gas_ + ∆Gsolv.

## Data Availability

The data presented in this study are available in this article.

## Supplementary Materials

The following are available online at https://www.mdpi.com/article/10.3390/biology10040319/s1: Figure S1. Effects of selected bioactive compounds and rice bran extracts on steroid 5α-reductase isoenzyme (*SRD5A*) expression in DU-145 cells treated with 0.10 mg/mL of ethanolic rice bran extracts (TRB, YRB, RRB, and MRB), 0.01 mg/mL of selected bioactive compounds, and 0.10 mg/mL of standard controls (finasteride and dutasteride). (a) Suppression of *SRD5A1*. (b) Suppression of *SRD5A2*. (c) Suppression of *SRD5A3*. A statistical significance in comparison to controls is indicated as * *p* < 0.05; Table S1. The percentage of *SRD5A* suppression of four rice bran extracts and their bioactive constituents.
