# Supplementary material for: Effects on Steroid 5-Alpha Reductase Gene Expression of Thai Rice Bran Extracts and Molecular Dynamics Study on SRD5A2
Source: Biology (Basel). 2021 Apr 11;10(4):319. doi: 10.3390/biology10040319 (PMC8070419; doi:10.3390/biology10040319)
Supplement: Supplementary file 1 [file biology-10-00319-s001.zip › biology-1158711 supplementary materials/biology-1158711 revised supplementary.docx]

Article

Effects on Steroid 5‐alpha Reductase Gene Expression of Thai Rice Bran Extracts and Molecular Dynamics Study on SRD5A2

**Supplementary Information**


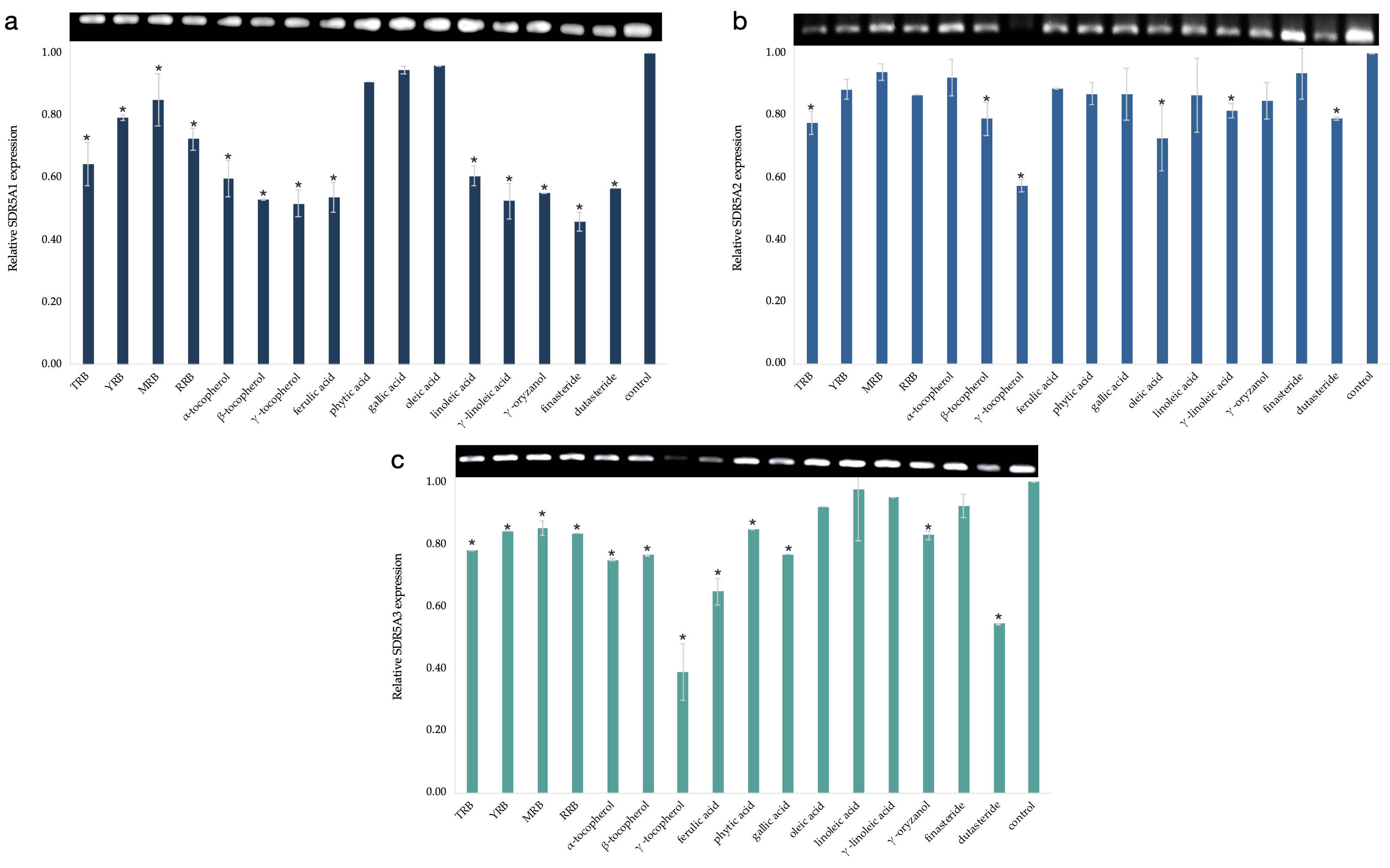


**Figure S1.** Effects of selected bioactive compounds and rice bran extracts on 5α-reductase isoenzymes (*SRD5As*) expression in DU-145 treated with the 0.10 mg/mL ethanolic rice bran extracts (TRB, YRB, RRB, and MRB), 0.01 mg/mL selected bioactive compounds, and 0.10 mg/mL standard controls (finasteride and dutasteride). (a) the suppression of *SRD5A1.* (b) the suppression of *SRD5A2.* (c) the suppression of *SRD5A3*. A statistical significance in comparison to control is indicated as * (*p* < 0.05).

**Table S1**. The percentage of *SRD5As* suppression of four rice bran extracts and their bioactive constituents

| **Sample** | ***SR5A1* suppression (%)** | | | ***SR5A2* suppression (%)** | | | ***SR5A3* suppression (%)** | | |
| --- | --- | --- | --- | --- | --- | --- | --- | --- | --- |
| TRB | 35.79 | ± | 6.94 | 22.26 | ± | 3.73 | 21.97 | ± | 0.01 |
| YRB | 20.66 | ± | 0.92 | 11.63 | ± | 3.17 | 15.86 | ± | 0.01 |
| MRB | 14.90 | ± | 8.37 | 6.09 | ± | 2.60 | 14.85 | ± | 2.26 |
| RRB | 27.61 | ± | 3.55 | 13.49 | ± | 0.00 | 16.55 | ± | 0.01 |
| α-Tocopherol | 40.31 | ± | 6.04 | 7.81 | ± | 5.93 | 24.98 | ± | 0.31 |
| β-Tocopherol | 47.01 | ± | 0.00 | 21.12 | ± | 5.44 | 23.45 | ± | 0.01 |
| γ-Tocopherol | 48.32 | ± | 4.29 | 42.57 | ± | 1.91 | 61.04 | ± | 9.10 |
| Ferulic acid | 46.33 | ± | 4.94 | 11.32 | ± | 0.22 | 35.22 | ± | 4.19 |
| Phytic acid | 9.28 | ± | 0.00 | 13.06 | ± | 3.63 | 15.25 | ± | 0.01 |
| Gallic acid | 5.45 | ± | 1.12 | 13.22 | ± | 8.36 | 23.34 | ± | 0.01 |
| Oleic acid | 4.16 | ± | 0.00 | 27.49 | ± | 10.42 | 7.99 | ± | 0.01 |
| Linoleic acid | 39.52 | ± | 3.17 | 13.49 | ± | 11.84 | 2.48 | ± | 16.30 |
| γ-Linolenic acid | 47.54 | ± | 5.66 | 18.48 | ± | 2.39 | 4.97 | ± | 0.01 |
| γ-Oryzanol | 45.07 | ± | 0.00 | 15.32 | ± | 5.80 | 17.11 | ± | 1.54 |
| Finasteride | 54.21 | ± | 3.05 | 6.48 | ± | 8.22 | 7.70 | ± | 3.58 |
| Dutasteride | 43.50 | ± | 0.01 | 20.84 | ± | 0.54 | 45.57 | ± | 0.03 |


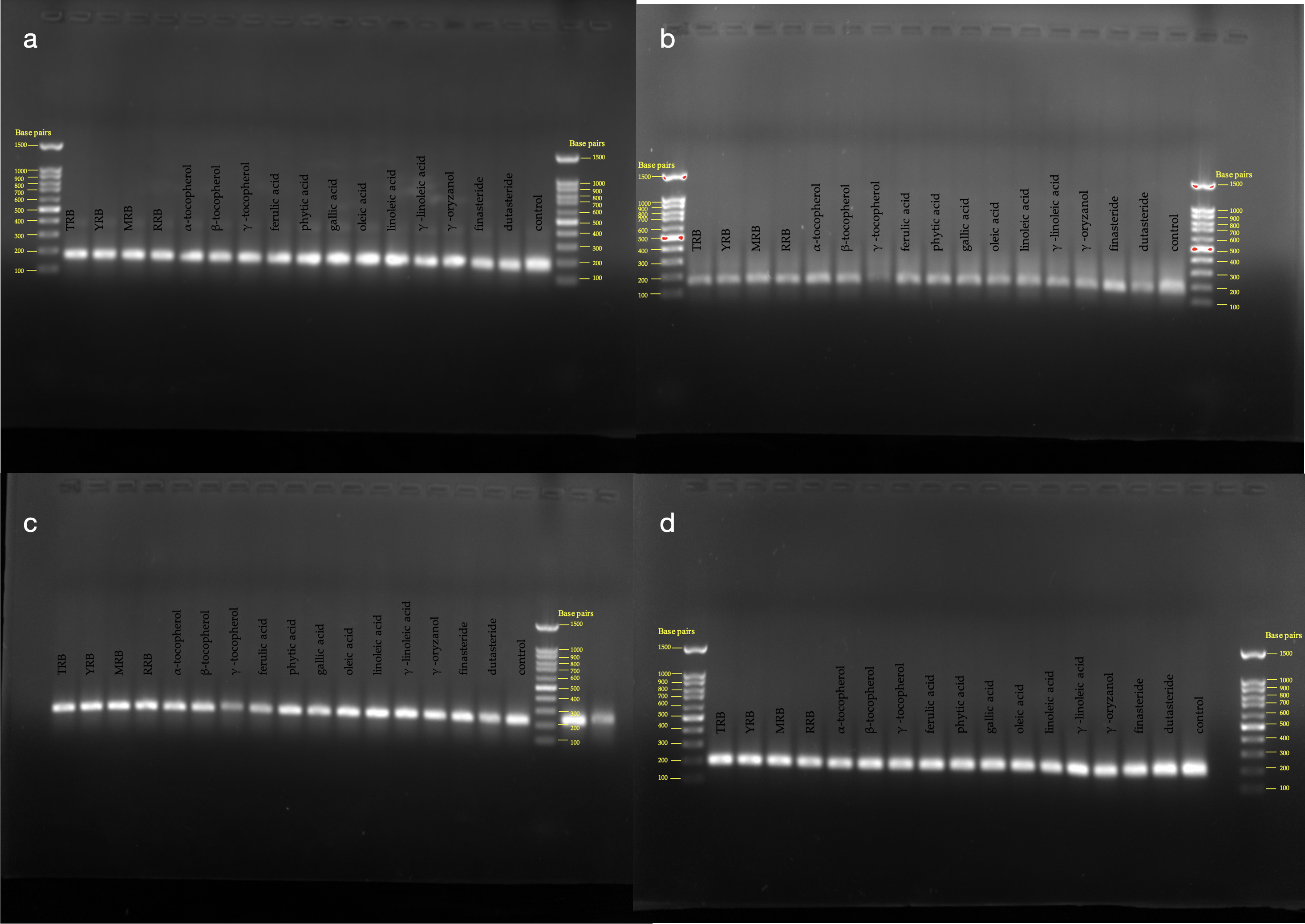


**Figure S2.** Original image of electrophoresis on 1% (*w/v*) agarose gel of (a) *SRD5A1* (estimated product length 163 base pairs), (b) *SRD5A2* (estimated product length 154 base pairs), (c) *SRD5A3* (estimated product length 211 base pairs), and (d) *GAPDH* (estimated product length 185 base pairs)
